# Association Between Base Excess and Mortality Among Patients in ICU With Acute Kidney Injury

**DOI:** 10.3389/fmed.2021.779627

**Published:** 2021-12-02

**Authors:** Yi Cheng, You Zhang, Boxiang Tu, Yingyi Qin, Xin Cheng, Ran Qi, Wei Guo, Dongdong Li, Shengyong Wu, Ronghui Zhu, Yanfang Zhao, Yuanjun Tang, Cheng Wu

**Affiliations:** ^1^Department of Military Health Statistics, Naval Medical University, Shanghai, China; ^2^Department of Nephrology, Changhai Hospital, Naval Medical University, Shanghai, China; ^3^Jinan People's Hospital Affiliated to Shandong First Medical University, Shandong, China; ^4^The Second Children & Women's Healthcare of Jinan City, Shandong, China; ^5^Department of Clinical Pharmacy, Shanghai General Hospital, School of Medicine, Shanghai Jiaotong University, Shanghai, China

**Keywords:** base excess, acute kidney injury, 30-day mortality, restrict cubic splines, MIMIC-IV

## Abstract

**Objective:** This study aimed to explore the association between base excess (BE) and the risk of 30-day mortality among patients with acute kidney injury (AKI) in the intensive care unit (ICU).

**Methods:** This retrospective study included patients with AKI from the Medical Information Mart for Intensive Care (MIMIC)-IV database. We used a multivariate Cox proportional-hazards model to obtain the hazard ratio (HR) for the risk of 30-day mortality among patients with AKI. Furthermore, we utilized a Cox proportional-hazard model with restricted cubic splines (RCS) to explore the potential non-linear associations.

**Results:** Among the 14,238 ICU patients with AKI, BE showed a U-shaped relationship with risk of 30-day mortality for patients with AKI, and higher or lower BE values could increase the risk. Compared with normal base excess (−3~3 mEq/L), patients in different groups (BE ≤ −9 mEq/L, −9 mEq/L < BE ≤ −3 mEq/L, 3 mEq/L < BE ≤ 9 mEq/L, and BE > 9 mEq/L) had different HRs for mortality: 1.57 (1.40, 1.76), 1.26 (1.14, 1.39), 0.97 (0.83, 1.12), 1.53 (1.17, 2.02), respectively. The RCS analyses also showed a U-shaped curve between BE and the 30-day mortality risk.

**Conclusion:** Our results suggest that higher and lower BE in patients with AKI would increase the risk of 30-day mortality. BE measured at administration could be a critical prognostic indicator for ICU patients with AKI and provide guidance for clinicians.

## Introduction

Acute kidney injury (AKI) is a severe complication in the intensive care unit (ICU), as it is associated with higher morbidity, mortality, and poorer outcomes. The incidence of AKI ranges from 2% in community settings to 20% among hospitalized patients and up to 60% in ICUs (1).

Patients with AKI show an increased risk of mortality, chronic kidney disease (CKD) progression and cardiovascular complications (2). And the mortality in hospitalized AKI patients is 10.8%. In addition, as diuretics, vasopressors, and renal replacement therapy (RRT) can be administered to these patients, these factors can lead to acid–base disturbances.

Base excess (BE) is defined as the amount of acid or alkali needed to adjust the value to the normal range (3), which is considered a pure indicator of metabolic acid–based balance (4). Base excess has been widely used as a predictor for the diagnosis of neonatal sepsis in pre-term newborns (5), complications in cytoreductive surgery (6), or the prognosis of neonatal acute respiratory distress syndrome (7). The indicator also shows significant representativeness in heart failure (3, 4). However, the association between BE and mortality among patients with AKI remains unclear.

Smith (8) demonstrated that BE could be a prognostic indicator for patients admitted to the ICU. Those patients with BE on admissions < −4 mmol/L had higher mortality than those with BE ≥ −4 mmol/L (57.7 vs. 17.6%, *P* < 0.0001). However, in this study, other confounders, such as age, sequential organ failure assessment (SOFA) score on admission, inotropes, and mechanical ventilation were significantly different between the two groups and were not adjusted.

Consequently, the aim of this study was to explore the effect of baseline BE on mortality among patients with AKI.

## Materials and Methods

### Source of Data

The data of this study were extracted from a large critical care database named Medical Information Mart for Intensive Care (MIMIC)-IV (9, 10), which is a publicly and freely available database. The MIMIC-IV database, an update to MIMIC-III, contained data for patients who were admitted to the Beth Israel Deaconess Medical Center (BIDMC) from 2008–2019. After completing the National Institutes of Health (NIH) web-based training course and the Protecting Human Research Participants examination, we extracted data from MIMIC-IV. An author who has completed the Collaborative Institutional Training Initiative examination (Certification Number 39090498 for author YY Qin) can access the database.

### Participants

This study included adult patients (≥18 years) with AKI defined according to the Kidney Disease Improving Global Outcomes (KDIGO) criteria (Table 1). The KDIGO criteria were described as follows (11): (1) Serum creatinine (SCr) increased by 0.3 mg/dL (or ≥26.5 μmol/L) within 48 h; or (2) increased by ≥1.5-fold from baseline within the prior 7 days; and/or (3) a decrease in urine output (UO) < 0.5 ml/kg/h for 6–12 h. AKI was diagnosed after ICU admission. Patients without BE values when admitted to the ICU and those who died or were discharged within 48 h after ICU admission were excluded. If a patient had multiple admission records or ICU stay records, we only took the first admission record and the first ICU stay record.

**Table 1:** AKI definition and classification according to the kidney disease improving global outcomes (KDIGO) criteria.

	**SCr criteria**	**UO criteria**
**AKI definition**	SCr increases by 0.3 mg/dL (or ≥26.5 μmol/L) in ≤ 48 h Or rises to ≥1.5-fold from baseline within the prior 7 days	And/or a decrease in UO <0.5 mL/kg/h for 6–12 h
**AKI classification**
Stage 1	SCr ≥0.3 rise within 48 h or SCr ≥1.5- to 1.9-fold rise from baseline (previous lowest value) within 7 days	<1 mL/kg/h for 24 h
Stage 2	SCr increase >2.0- to 2.9-fold from baseline	<0.5 mL/kg/h for ≥24 h
Stage 3	SCr increase >3.0-fold from baseline or serum creatinine ≥2.5 mg/dL(221 μmol/L) or initiation of RRT	<0.3 mL/kg/h for 24 h

### Variables

The variable of interest is baseline BE. Thus, we extracted the first measurement of BE at ICU admission as the baseline BE value. This was the main exposure factor in our study.

Other variables were extracted from the MIMIC-IV database according to the first day of ICU admission as potential confounding factors, including age at the time of hospital admission, admission type, sex, ethnicity, lactate, measure of acidity or alkalinity (pH), partial pressure of oxygen (PO_2_), and partial pressure of carbon dioxide (PCO_2_). AKI stages were defined by both SCr and the volume of Urine Output (UO) within the first 48 h after ICU admission according to KDIGO criteria (Table 1), comorbidities, simplified Acute Physiology Score II (SAPAII), SOFA score, SCr level, UO, use of vasopressors, renal replacement therapy (RRT), and mechanical ventilation. All variables were extracted from MIMIC-IV using postgreSQL.

### Endpoints

The primary endpoint was 30-day ICU mortality, which was defined by patient survival status at the time of hospital discharge. ICU mortality, in-hospital mortality, ICU length of stay (LOS), and hospital LOS were regarded as secondary endpoints.

### Missing Data

In the present study, all variables had missing values of <20%. We used multiple imputation (12) to impute missing values in variables, including lactate, urine output, and creatinine.

### Statistical Analysis

Descriptive statistics were used to present the differences between characteristics across the five groups of baseline BE values (BE ≤ −9 mEq/L, −9 mEq/L < BE ≤ −3 mEq/L, −3 mEq/L < BE ≤ 3 mEq/L, 3 mEq/L < BE ≤ 9 mEq/L, and BE > 9 mEq/L). Continuous variables were presented as mean ± standard deviation or median (IQR), and the differences between groups were identified using analysis of variance (ANOVA) or Kruskal–Wallis test. Categorical variables were presented as percentages, and comparisons between groups were made using the chi-square test or Fisher's exact-test.

The Cox proportional hazards model was used to estimate the association between predefined groups according to baseline BE values and outcomes among critically ill patients with AKI. The model was adjusted by multiple covariates with a *P*-value < 0.05, in univariate analysis. To assess the robustness of the results, which could be affected by potential unmeasured confounders, we calculated the *E*-value. The *E*-value was defined as the minimum strength of association that an unmeasured confounder would have between the explaining variable and the outcome (13) and the equation of *E*-value was: *E*-value = *HR*+*sqr*{*HR*× (*HR*−1)}.

In view of the hypothesis that the relationship between BE and risk of 30-day hospital mortality was non-linear, we further examined Cox models with restricted cubic splines (RCS) (14–16), with five knots (10th, 25th, 50th, 75th, and 90th percentiles) for BE, adjusting for all covariates above to flexibly model the association of baseline BE values with mortality. In addition, we used a likelihood ratio test to examine the non-linearity by comparing the model with only the linear term and with linear and cubic spline terms.

All statistical analyses were performed using the R software (version 4.0.0). All reported *P*-values were two-sided, and statistical significance was set at *P* < 0.05.

## Results

**Figure 1:**
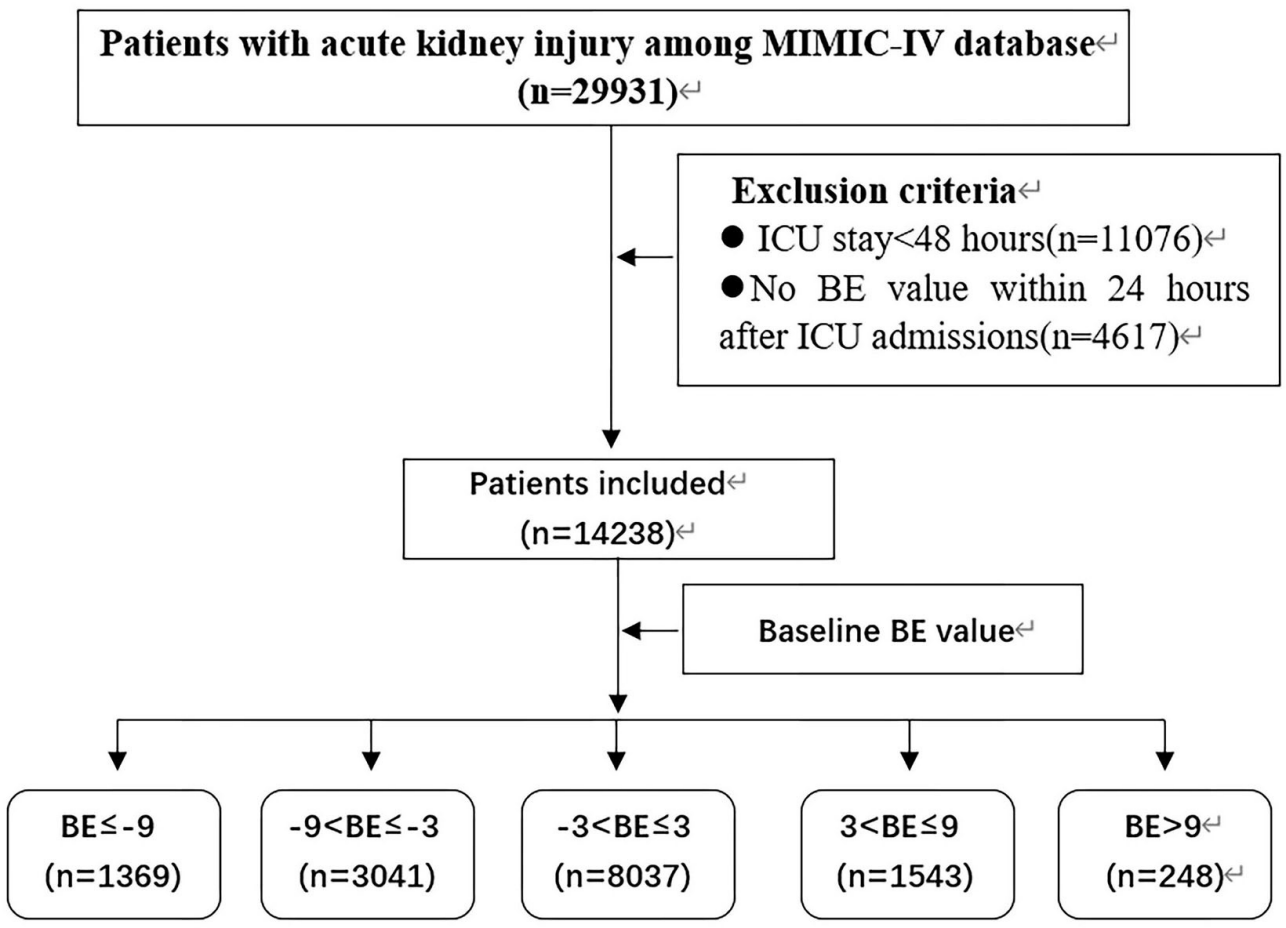
Flow chart of patient selection.

A total of 18,855 patients fulfilled the definition of AKI. Of these 14,238 patients whose ICU time was more than 2 days had a BE value within 24 h after ICU admission. The study flow diagram is shown in Figure 1.

Baseline characteristics of the five BE groups are depicted in Table 2. Compared with the normal group (−3 mEq/L < BE ≤ 3 mEq/L), those with lower BE values tended to be female, younger, and the values of lactate, creatinine, SOFA score, and SAPSII were higher, while the pH, PO_2_, PCO_2_, and UO were lower. The proportion of patients using vasopressors and RRT was also higher. Patients with higher BE values tended to be female, older, and had lower lactate and PO_2_ values. These patients had higher values of pH, PCO_2_, SOFA, and SAPSII, tended to use fewer vasopressors and ventilation, and were more likely to have comorbidities such as congestive heart failure and chronic pulmonary disease.

**Table 2:** Baseline characteristics between BE groups.

	**BE ≤ −9**	**−9 < BE ≤−3**	**−3 < BE ≤ 3**	**3 < BE ≤ 9**	**BE > 9**	***P-*value**
	***N = 1,369***	***N = 3,041***	***N = 8,037***	***N = 1,543***	***N = 248***	
**Gender: male**	747 (54.6%)	1,762 (57.9%)	4,834 (60.1%)	812 (52.6%)	106 (42.7%)	<0.001
**Age**	62.4 (17.1)	64.8 (17.3)	67.5 (15.1)	68.8 (14.9)	69.6 (14.5)	<0.001
**AKI stage:**						<0.001
1	170 (12.4%)	502 (16.5%)	1,772 (22.0%)	283 (18.3%)	45 (18.1%)	
2	427 (31.2%)	1,324 (43.5%)	4,267 (53.1%)	799 (51.8%)	127 (51.2%)	
3	772 (56.4%)	1,215 (40.0%)	1,998 (24.9%)	461 (29.9%)	76 (30.6%)	
**Ethnicity**						<0.001
BLACK	152 (11.1%)	255 (8.39%)	560 (6.97%)	143 (9.27%)	22 (8.87%)	
OTHER	443 (32.4%)	911 (30.0%)	2,027 (25.2%)	351 (22.7%)	63 (25.4%)	
WHITE	774 (56.5%)	1,875 (61.7%)	5,450 (67.8%)	1,049 (68.0%)	163 (65.7%)	
**Admission type**						<0.001
Elective	137 (10.0%)	384 (12.6%)	1,380 (17.2%)	241 (15.6%)	26 (10.5%)	
Emergency	919 (67.1%)	1,805 (59.4%)	3,515 (43.7%)	693 (44.9%)	141 (56.9%)	
Emergency surgery	37 (2.70%)	186 (6.12%)	1,312 (16.3%)	155 (10.0%)	7 (2.82%)	
Urgent	276 (20.2%)	666 (21.9%)	1,830 (22.8%)	454 (29.4%)	74 (29.8%)	
Lactate	5.19 (3.95)	2.69 (2.34)	1.72 (0.96)	1.46 (0.77)	1.29 (1.20)	0.000
pH	7.17 (0.12)	7.31 (0.07)	7.39 (0.07)	7.43 (0.08)	7.42 (0.11)	0.000
PO_2_	163 (113)	166 (115)	219 (142)	200 (141)	132 (114)	<0.001
PCO_2_	40.2 (16.0)	41.0 (11.5)	41.9 (10.6)	48.1 (15.9)	67.6 (23.3)	<0.001
Urine output	1,409 (1,588)	1,595 (1,235)	1,734 (1,098)	1,716 (1,227)	2,009 (1,540)	<0.001
SOFA	10.8 (4.20)	8.36 (4.01)	6.39 (3.39)	6.40 (3.38)	6.48 (3.40)	0.000
SAPSII	51.8 (15.6)	45.2 (15.0)	39.3 (13.0)	39.6 (12.3)	40.1 (12.9)	<0.001
Creatinine	2.55 (2.46)	1.88 (1.93)	1.24 (1.14)	1.32 (1.41)	1.19 (1.06)	<0.001
RRT	219 (16.0%)	197 (6.48%)	274 (3.41%)	88 (5.70%)	7 (2.82%)	<0.001
Vasopressor	286 (20.9%)	249 (8.19%)	344 (4.28%)	60 (3.89%)	7 (2.82%)	<0.001
Ventilation	348 (25.4%)	1,074 (35.3%)	3,651 (45.4%)	696 (45.1%)	102 (41.1%)	<0.001
**Co-morbidities**
Myocardial infarct	306 (22.4%)	640 (21.0%)	1,582 (19.7%)	284 (18.4%)	34 (13.7%)	0.003
Congestive heart failure	395 (28.9%)	941 (30.9%)	2,478 (30.8%)	711 (46.1%)	144 (58.1%)	<0.001
Cerebrovascular disease	161 (11.8%)	431 (14.2%)	1,532 (19.1%)	224 (14.5%)	24 (9.68%)	<0.001
Chronic pulmonary disease	327 (23.9%)	743 (24.4%)	2,146 (26.7%)	627 (40.6%)	153 (61.7%)	<0.001
Peptic ulcer disease	61 (4.46%)	104 (3.42%)	159 (1.98%)	29 (1.88%)	7 (2.82%)	<0.001
Mild liver disease	363 (26.5%)	543 (17.9%)	878 (10.9%)	161 (10.4%)	18 (7.26%)	<0.001
diabetes	453 (33.1%)	957 (31.5%)	2,430 (30.2%)	530 (34.3%)	88 (35.5%)	0.005
Renal disease	345 (25.2%)	767 (25.2%)	1,558 (19.4%)	370 (24.0%)	46 (18.5%)	<0.001
Malignant cancer	161 (11.8%)	443 (14.6%)	953 (11.9%)	176 (11.4%)	28 (11.3%)	0.002
Severe liver disease	177 (12.9%)	262 (8.62%)	413 (5.14%)	80 (5.18%)	5 (2.02%)	<0.001
Metastatic solid tumor	70 (5.11%)	197 (6.48%)	393 (4.89%)	97 (6.29%)	13 (5.24%)	0.009
AIDS	11 (0.80%)	21 (0.69%)	31 (0.39%)	2 (0.13%)	2 (0.81%)	0.010

*The rates of missing values for lactate, urine output, and creatinine are 11.72%, 1.55%, 0.01%, respectively*.

Table 3 shows the association between BE and all-cause mortality in patients with AKI. In the unadjusted model (Model 1), compared with the normal group, the hazard ratios [95% confidence interval (CI)] of the first, second, fourth, and fifth baseline BE groups were 1.57 (1.40, 1.76), 1.26 (1.14, 1.39), 0.97 (0.83, 1.12), 1.53 (1.17, 2.02), respectively. In the multivariable adjusted model (Model 3), the baseline BE showed a U-shaped association with 30-day all-cause mortality in the ICU. In model 3, the third group (−3 mEq/L < BE ≤ 3 mEq/L) had the lowest HR, and the fifth group had the highest HR (1.54, 95% CI: 1.16, 2.05). The HR (95% CI) of the first, second, and fourth group were 1.29 (1.13, 1.47), 1.12 (1.01, 1.24), 1.00 (0.86, 1.17), respectively. The result of model 3 was showed in the Supplementary Table S1.

**Table 3:** HR of 30-day hospital mortality according to BE in patients with AKI.

**BE value**	***N***	**Events**	**Hazard ratio (95%CI)**					
			**Model 1**	***P***	**Model 2**	***P***	**Model 3**	***P***
BE ≤ −9	1,369	389	1.57 (1.40, 1.76)	<0.01	1.71 (1.53, 1.92)	<0.01	1.29 (1.13, 1.47)	<0.01
−9 < BE ≤ −3	3,041	584	1.26 (1.14, 1.39)	<0.01	1.31 (1.19, 1.45)	<0.01	1.12 (1.01, 1.24)	0.04
−3 < BE ≤ 3	8,037	965	Reference					
3 < BE ≤ 9	1,543	197	0.97 (0.83, 1.12)	0.64	0.94 (0.81, 1.09)	0.43	1.00 (0.86, 1.17)	0.99
BE > 9	248	54	1.53 (1.17, 2.02)	<0.01	1.46 (1.11, 1.92)	<0.01	1.54 (1.16, 2.05)	<0.01

*Model 1: unadjusted model. Model 2: adjusted for age and gender. Model 3: adjusted by gender, age, ethnicity, AKI stage, PCO_2_, Co-morbidities, urine output, SOFA, SAPSII, RRT, vasopressor, ventilation, Creatinine*.

According to the equation, *E*-value was obtained as follows:


(1) $$\begin{array}{r} E-value=1.57+sqr\left\{1.57\times \left(1.57-1\right)\right\}=2.52 \end{array}$$

In our study, the *E*-value was relatively large; thus, we can consider the results to be robust to some extent.

**Figure 2:**
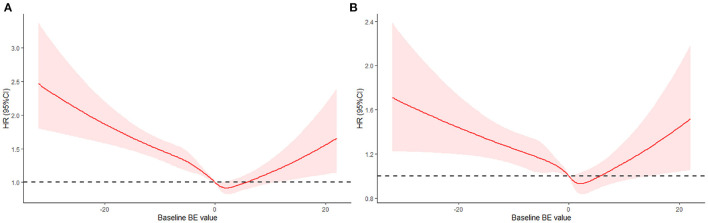
Hazard ratio of all-cause mortality as a function of baseline BE value. **(A)** Baseline BE was modeled as a continuous variable and fitted in an unadjusted model using restricted quadratic spline regression. **(B)** Baseline BE was modeled as a continuous variable and adjusted by gender, age, ethnicity, AKI stage, PCO_2_, Co-morbidities, urine output, SOFA, SAPSII, RRT, vasopressor, ventilation, Creatinine.

Furthermore, we used RCS to flexibly model and visualize the relationship between baseline BE values and 30-day all-cause mortality in the ICU. In the restricted quadratic spline regression model, baseline BE was taken as a continuous variable. In Figure 2A, the univariate analysis showed that the HR of 30-day all-cause mortality in the ICU decreased to 1 and became steeper when the baseline BE value exceeded −5 mEq/L. We observed the lowest HR when the baseline BE value was 3 mEq/L and the risk started to increase afterwards (*P* for non-linearity < 0.01). We can also conclude that the baseline BE value in the range of 0–5 mEq/L was a protective factor (HR <1). When the BE value was outside this range, it was a risk factor (HR > 1). The results of the multivariate analysis (Figure 2B) were similar. The risk of mortality decreased and reached the lowest HR when the BE was 3 mEq/L, and then started to increase afterwards (*P* for non-linearity < 0.01).

The results of the secondary endpoints are presented in Tables 4, 5. The risk of ICU mortality and the risk of in-hospital mortality also showed U-shaped relationships. The median ICU LOS and hospital LOS were significantly different between the groups. Patients in the normal group (−3 mEq/L < BE ≤ 3 mEq/L) showed the shortest ICU LOS and hospital LOS (4.07 and 8.36 days, respectively). Both lower and higher BEs would increase the ICU LOS or hospital LOS. Multiple linear regression results showed that (Table 5), compared with the normal group, the first, second, and fourth groups increased the ICU LOS by 0.65, 0.48, and 0.41 days, respectively. As for hospital LOS, the first group would increase by 0.75 days. The other groups showed no significant differences.

**Table 4:** Multiple cox regression^*^ result for secondary outcomes according to BE groups in patients with AKI.

**BE value**	**ICU mortality**			**In-hospital mortality**		
	***N* (event)**	HR (95%CI)^*^	***P***	***n* (event)**	HR (95%CI)^*^	***P***
BE ≤ −9	1,369 (376)	1.33 (1.15, 1.52)	<0.01	1,369 (426)	1.27 (1.12, 1.44)	<0.01
−9 < BE ≤ −3	3,041 (546)	1.16 (1.04, 1.30)	<0.01	3,041 (658)	1.10 (1.00, 1.22)	0.06
−3 < BE ≤ 3	8,037 (858)	Reference		8,037 (1,093)	Reference	
3 < BE ≤ 9	1,543 (174)	0.98 (0.83, 1.16)	0.85	1,543 (216)	1.02 (0.88, 1.18)	0.83
BE > 9	248 (43)	1.43 (1.04, 1.97)	0.03	248 (55)	1.60 (1.21, 2.12)	<0.01

* *Adjusted by gender, age, ethnicity, AKI stage, PCO_2_, Co-morbidities, urine output, SOFA, SAPSII, RRT, vasopressor, ventilation, creatinine*.

**Table 5:** Multiple linear regression^*^ results examining the effect of BE in patients with AKI.

**BE value**	***N***	**LOS of ICU**			**LOS of hospital**		
		Median (IQR)^**^	**β**	***P-*value**	Median (IQR)^**^	**β**	***P-*value**
BE ≤ −9	1,369	6.12 (3.54, 11.0)	0.65	<0.01	11.8 (6.47, 20.40)	0.75	<0.01
−9 < BE ≤ −3	3,041	5.03 (3.14, 9.31)	0.48	<0.01	10.9 (6.61, 17.90)	1.23	0.06
−3 < BE ≤ 3	8,037	4.07 (2.80, 7.21)	Reference		8.36 (5.65, 14.60)	Reference	
3 < BE ≤ 9	1,543	4.64 (2.95, 8.16)	0.41	0.03	9.07 (6.14, 15.00)	0.01	0.78
BE > 9	278	4.21 (2.96, 8.82)	−0.15	0.74	9.35 (5.80, 14.70)	−0.03	0.98

* *Adjusted by gender, age, ethnicity, AKI stage, PCO_2_, Co-morbidities, urine output, SOFA, SAPSII, RRT, vasopressor, ventilation, Creatinine*.

** *By Kruskal-Wallis H-test, P-value < 0.05*.

### Subgroup Analysis

**Figure 3:**
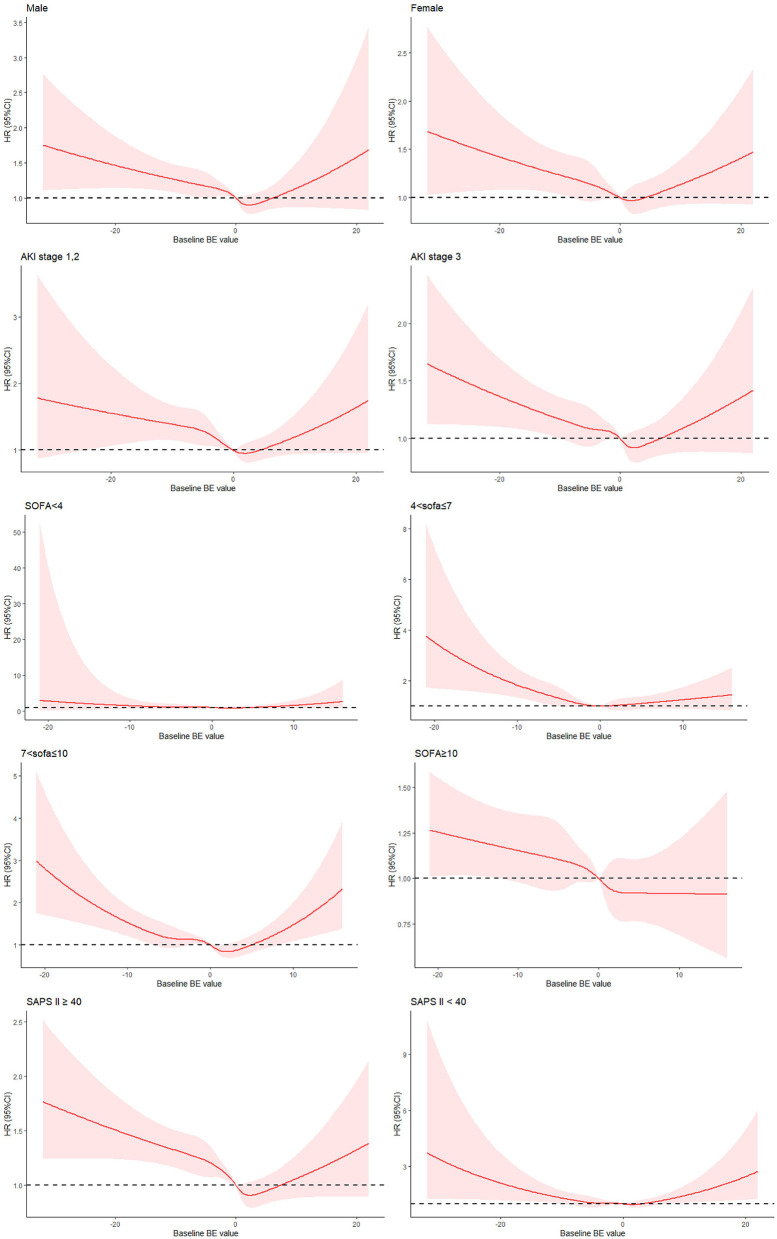
Risk of all-cause mortality according to BE among four subgroups.

Subgroup analysis was based on the following strata: sex (female vs. male), AKI stage (stage 3 vs. stages 1, 2), SOFA score (SOFA ≥ 10, 7 ≤ SOFA <10, 4 ≤ SOFA <7, SOFA <4), and SAPS II score. We divided the patients into four subgroups according to SOFA score quartiles. We also divided the patients into two groups according to the median SAPS II score (median = 40). In almost all subgroups, when BE was in the range of −3~9 mEq/L, the patients had the lowest risk for mortality, and the patients in the fifth BE group (BE > 9 mEq/L) had the highest risk (Table 6). Similar trends of non-linear association between BE and all-cause mortality are presented in Figure 3. After using the multiple Cox model with RCS, BE showed a U-shaped relationship with all-cause mortality in most subgroups (except the SOFA ≥ 10 subgroup). The results of the subgroup analysis for secondary endpoints are shown in Supplementary Tables S2–S5.

**Table 6:** HR of 30-day ICU mortality according to BE among subgroups.

**Subgroups**	**Hazard ratio (95%CI)**				
	**BE ≤ –9 mEq/L**	**–9 < BE ≤ –3 mEq/L**	**–3 < BE ≤ 3 mEq/L**	**3 < BE ≤ 9 mEq/L**	**BE > 9 mEq/L**
**Male**
*N* (event)	747 (215)	1,762 (342)	4,834 (573)	812 (99)	106 (21)
Adjusted HR (95%CI)	1.27 (1.07, 1.52)	1.12 (0.97, 1.29)	Reference	1.01 (0.81, 1.26)	1.36 (0.87, 2.12)
*P-*value	<0.01	0.12		0.93	0.17
**Female**
*N* (event)	622 (190)	1,279 (272)	3,203 (457)	731 (107)	142 (33)
Adjusted HR (95%CI)	1.30 (1.07, 1.58)	1.11 (0.95, 1.30)	Reference	0.98 (0.79, 1.22)	1.66 (1.14, 2.42)
*P-*value	<0.01	0.20		0.88	<0.01
**AKI stage is 1, 2**
*N* (event)	597 (107)	1,826 (227)	6,039 (516)	1,082 (102)	172 (24)
Adjusted HR (95%CI)	1.39 (1.10, 1.74)	1.20 (1.02, 1.41)	Reference	1.08 (0.87, 1.35)	1.45 (0.94, 2.24)
*P-*value	<0.01	0.03		0.47	0.09
**AKI stage is 3**
*N* (event)	772 (298)	1,215 (387)	1,998 (514)	461 (104)	76 (30)
Adjusted HR (95%CI)	1.23 (1.05, 1.44)	1.07 (0.93, 1.23)	Reference	0.94 (0.76, 1.16)	1.67 (1.14, 2.43)
*P-*value	0.01	0.32		0.55	<0.01
**SOFA** **≥** **10**
*N* (event)	747 (215)	1,762 (342)	4,834 (573)	812 (99)	106 (21)
Adjusted HR (95%CI)	1.16 (0.99, 1.37)	1.12 (0.97, 1.31)	Reference	0.74 (0.55, 0.99)	1.18 (0.72, 1.94)
*P-*value	0.07	0.12		0.04	0.51
**7** **≤** **SOFA** **<** **10**
*N* (event)	280 (78)	839 (138)	2,084 (301)	392 (64)	66 (18)
Adjusted HR (95%CI)	1.78 (1.37, 2.32)	1.11 (0.91, 1.37)	Reference	1.10 (0.84, 1.46)	2.10 (1.27, 3.48)
*P-*value	<0.01	0.33		0.47	<0.01
**4** **≤** **SOFA** **<** **7**
*N* (event)	191 (25)	788 (102)	2,992 (264)	570 (69)	90 (11)
Adjusted HR (95%CI)	2.16 (1.40, 3.31)	1.33 (1.05, 1.69)	Reference	1.26 (0.96, 1.67)	1.41 (0.73, 2.70)
*P-*value	<0.01	0.02		0.10	0.30
**SOFA2** **<** **4**
*N* (event)	53 (3)	303 (20)	1,592 (106)	307 (20)	45 (8)
Adjusted HR (95%CI)	1.93 (0.59, 6.30)	1.29 (0.78, 2.11)	Reference	1.06 (0.64, 1.77)	3.66 (1.55, 8.62)
*P-*value					
**SAPS II** **≥** **40**
*N* (event)	1,066 (367)	1,895 (511)	3,584 (695)	719 (125)	115 (37)
Adjusted HR (95%CI)	1.33 (1.16, 1.53)	1.18 (1.05, 1.33)	Reference	0.89 (0.73, 1.08)	1.51 (1.07, 2.12)
*P-*value	<0.01	<0.01		0.23	0.02
**SAPS II** **<** **40**
*N* (event)	303 (38)	1,146 (103)	4,453 (335)	824 (81)	133 (17)
Adjusted HR (95%CI)	1.48 (1.03, 2.11)	1.07 (0.85, 1.34)	Reference	1.27 (0.99, 1.64)	1.89 (1.12, 3.20)
*P-*value	0.03	0.58		0.06	0.02

## Discussion

Base excess was considered to be one of the most widely used markers in ICU, both to diagnose metabolic acidosis and to guide resuscitation or clinical intervention (17). In this study, we explored the U-shaped relationship between BE and all-cause mortality in ICU patients with AKI. Patients with AKI in the ICU had lower risk for mortality when BE was in the range of −3~9 mEq/L. Both higher and lower BEs would increase the risk.

BE is convenient and intuitive for reflecting acid–base status in the ICU setting. Many studies have focused on base excess as one of the most essential indicators in ICU settings. For patients with trauma, the use of BE appeared to be better than vital signs or shock index for mortality prediction or rapid physiological assessment (18). A systematic review involving 34 studies on trauma patients consistently showed that BE was associated with increased mortality, significant injuries, and major complications (19).

The use of BE has also been extensively studied in burns. A prospective study including 42 major burn patients suggested that base deficit (BD, which is equal to -BE), as well as serum albumin level and hemoglobin concentration, were independent predictors of mortality among patients with burns (20). Binary logistic regression analysis revealed that the odds ratio of BD was 2.23 (95% CI: 1.66–16.75). Furthermore, BE has also been used in newborns and children, for example, in the prediction of early diagnosis of neonatal sepsis.

The vital role of BE in prognosis and prediction has been demonstrated in many diseases. To determine the prognostic value of BE on admission among patients with acute heart failure (AHF), Hiroki Nakano et al. (4) concluded that high BE was an independent determinant of all-cause death in patients with AHF. Similarly, the unadjusted spline curve revealed a U-shaped relationship between BE and the risk of all-cause death. In the multivariable RCS model, a positive linear relationship was observed. Another study also showed a U-shaped association between BE and survival outcomes among patients with congestive heart failure (3).

As for AKI, the relationship between BE and all-cause mortality was more complex. The kidney plays a vital role in maintaining electrolyte homeostasis and acid–base balance. Acid–base disorder is not only a consequence, but also a contributor to the progression of kidney dysfunction.

The U-shaped curve showed that lower BE (≤ -3 mEq/L), which indicated metabolic acidosis, would increase the risk of all-cause mortality in patients with AKI. This result was consistent with another study (21) which suggested that metabolic acidosis was an independent risk factor for the progression of AKI and hospital mortality. This may be because acidosis can reduce renal blood flow and increase inflammatory mediator release (22). Another study (23) demonstrated that hyperchloremic acidosis was associated with post-operative AKI after abdominal surgery. Patients with AKI with severe metabolic alkalosis (BE > 9 mEq/L) had a higher risk of all-cause death, as shown in the U-shaped curve. Significant metabolic alkalosis is rare in AKI and is related to diuretic use, red blood cells and fresh frozen plasma administration (24).

The results of subgroup analysis according to SOFA or SAPSII showed that the risk of mortality monotonically decreased when SOFA > 10, and the risk of mortality was also U-shaped when 4 < SOFA <7, 7 < SOFA <10, SAPS II ≥ 40, and SAPS <40. When the SOFA score was <4, there was no significant U-shaped relationship (Figure 3). Some studies have recommended that organ dysfunction can be identified as an acute change in the total SOFA score ≥ 2 points (25, 26). Several studies also showed that patients with SAPS II > 80 had a higher survival rate (27, 28). However, if we divided patients into subgroups by SOFA (≥2 or <2) and SAPS II (>80 or ≤ 80), there would be fewer members in one group, leading to inaccurate results. Therefore, we divided the subgroups according to the median or quartiles. AKI stage was also a crucial factor for mortality among patients with AKI. In view of the differences in mortality among patients with different AKI stages in univariate analysis, we did subgroup analysis according to AKI stages. And U-shape relationship between BE and mortality among patients with AKI was also existed.

This study had several limitations. First, BE remains a convenient and sensitive indicator for metabolic acid–base status, but it is not consistent with other markers such as lactate, bicarbonate, or anion gap (29). Thus, clinicians should be careful and consider these markers.

In addition, BE can be severely influenced by previous or concomitant administration of bicarbonate or lactate during resuscitation and in-hospital stay before ICU admission. The proportion of bicarbonate and lactate was shown in Supplementary Table S6. We included the two variables into multivariable adjusted model and as part of the sensitivity analysis, we examined multiple Cox models with RCS. The results were showed in Supplementary Table S7 and Supplementary Figure S1, respectively. After including the variables of bicarbonate and lactate, the results changed slightly, but the statistical significance and overall conclusions remain the same. We considered that the low before ICU administration rate of bicarbonate and lactate might not affect the primary results substantially.

Furthermore, AKI can influence the acid–base imbalance; in turn, acid–base status can also be the result of therapies for deteriorating renal function, such as fluid resuscitation, use of diuretics, and RRT. Thus, for prerenal AKI, BE was appropriate for measuring the acid–base status. In contrast, BE had minimal utility for intrarenal or post-postrenal AKI. For example, when patients are on RRT in the ICU setting, BE only reflects the concentration of bicarbonate buffer and the duration of treatment. We considered that the low before ICU administration rate of bicarbonate and lactate might not affect the primary results substantially.

Finally, we considered the first measurement of BE at administration, regardless of the influence of dynamic variation in BE. In the follow-up study, we built a joint model for longitudinal and time-to-event data (30, 31) to control measurement error and explore the effect of BE variation on the risk of all-cause death in patients with AKI.

## Conclusions

BE could be an independent risk factor for all-cause death in patients with AKI in the ICU setting. Abnormal BE may be a risk factor for these patients. We also found a U-shaped association between baseline BE and all-cause mortality in patients with AKI. Regardless of whether BE is lower (BE ≤ −3 mEq/L) or higher (BE ≥ 9 mEq/L), it can increase the risk of 30-day ICU mortality in patients with AKI. Thus, BE measured at administration could be a good indicator of the severity of illness and provide guidance for clinicians.

## Data Availability Statement

The raw data supporting the conclusions of this article will be made available by the authors, without undue reservation.

## Author Contributions

YC, YQ, YoZ, and CW designed the study and wrote the manuscript. YT, BT, WG, and XC performed the statistical analysis. RQ, DL, YaZ, SW, and RZ collected the study data. All authors participated in writing and revising the manuscript and read and approved the final manuscript.

## Funding

This work was supported by Three-Year Action Plan for Strengthening Public Health System in Shanghai (2020-2022) Subject Chief Scientist (No.GWV-10.2-XD05); Military logistics scientific research project (No. AWS14R013-1); Military Key Disciplines Construction Project-03; Natural Science Foundation of Shanghai (No.19ZR1469800); The National Natural Science Foundation of China (No. 82003558).

## Conflict of Interest

The authors declare that the research was conducted in the absence of any commercial or financial relationships that could be construed as a potential conflict of interest.

## Publisher's Note

All claims expressed in this article are solely those of the authors and do not necessarily represent those of their affiliated organizations, or those of the publisher, the editors and the reviewers. Any product that may be evaluated in this article, or claim that may be made by its manufacturer, is not guaranteed or endorsed by the publisher.

## Abbreviations

AKI: acute kidney injury
ANOVA: Analysis of variance
BE: base excess
CKD: chronic kidney disease
ICU: intensive care unit
KDIGO: the Kidney Disease Improving Global Outcomes
pH: measure of acidity or alkalinity
PCO_2_: partial pressure of carbon dioxide
PO_2_: partial pressure of oxygen
RCS: restrict cubic splines
RRT: renal replacement therapy
SAPSII: simplified Acute Physiology Score II
SOFA: sequential organ failure assessment
LOS: length of stay
UO: urine output.
