# Supplementary material for: Association Between Base Excess and Mortality Among Patients in ICU With Acute Kidney Injury
Source: Front Med (Lausanne). 2021 Dec 2;8:779627. doi: 10.3389/fmed.2021.779627 (PMC8674681; doi:10.3389/fmed.2021.779627)
Supplement: Supplementary file 2 [file Data_Sheet_2.docx]

| **Table S1. The result of multivariable adjusted model (model3)** | | | |
| --- | --- | --- | --- |
| **Variables** | **Coefficient** | **HR (95% confidence interval)** | **P** |
| **Group** |  |  |  |
| Group1 | 0.253 | 1.29(1.13,1.47) | <0.01 |
| Group2 | 0.112 | 1.12(1.01,1.24) | 0.04 |
| Group 3 | Reference | | |
| Group4 | 0.001 | 1.00(0.86,1.17) | 0.99 |
| Group5 | 0.435 | 1.54(1.16,2.05) | <0.01 |
| **Gender(man)** | 0.010 | 0. 91(0.83,0.99) | 0.02 |
| **Age** | 0.019 | 1.02(1.01,1.02) | <0.01 |
| **Ethnicity** |  |  |  |
| Black | Reference | | |
| white | 0.050 | 1.05(0.98,1.24) | 0.55 |
| Other | 0.339 | 1.40(1.19,1.66) | <0.01 |
| **AKI stage** |  |  |  |
| Stage 1 | Reference | | |
| Stage2 | -0.154 | 0.86(0.73,1.00) | 0.05 |
| Stage3 | 0.131 | 1.14(0.98,1.33) | 0.10 |
| **PCO_2_** | 0.000 | 1.00(1.00,1.01) | 0.59 |
| **Co-morbidities** |  |  |  |
| Myocardial infarct | 0.173 | 1.19(1.07,1.32) | <0.01 |
| Congestive heart failure | 0.050 | 1.05(0.96,1.15) | 0.30 |
| Cerebrovascular disease | 0.252 | 1.29(1.17,1.42) | <0.01 |
| Chronic pulmonary disease | 0.044 | 0.96(0.87,1.05) | 0.36 |
| diabetes | -0.179 | 0.84(0.76,0.92) | <0.01 |
| Malignant cancer | 0.295 | 1.34(1.20,1.50) | <0.01 |
| Severe liver disease | 0.519 | 1.68(1.47,1.93) | <0.01 |
| **urine output** | 0.000 | 1.00(0.99,1.00) | 0.12 |
| **SOFA** | -0.009 | 0.99(0.98,1.01) | 0.24 |
| **SAPSII** | 0.016 | 1.02(1.01,1.02) | <0.01 |
| **RRT** | -0.119 | 0.89(0.75,1.05) | 0.14 |
| **vasopressor** | -0.105 | 0.90(0.78,1.04) | 0.153 |
| **ventilation** | -0.196 | 0.82(0.78,1.04) | <0.01 |
| **Creatinine** | 0.011 | 1.01(0.98,1.04) | 0.50 |

**Supplement Material**

| **Table S2. Adjusted HR of ICU mortality according to BE among Subgroups** | | | | | |
| --- | --- | --- | --- | --- | --- |
| **Subgroups** | **BE groups(mEq/L)** | | | | |
|  | **BE≤-9** | **-9<BE≤-3** | **-3<BE≤3** | **3<BE≤9** | **BE>9** |
| **Male** |  |  |  |  |  |
| HR (95%CI) | 1.35（1.12,1.63） | 1.16（0.99,1.34） | Reference | 0.99（0.78,1.26） | 1.35（0.84,2.18） |
| P value | <0.01 | 0.07 |  | 0.97 | 0.22 |
| **Female** |  |  |  |  |  |
| HR (95%CI) | 1.29(1.05,1.58) | 1.15(0.98,1.36) | Reference | 0.96(0.76,1.21) | 1.52(0.99,2.33) |
| P value | 0.02 | 0.09 |  | 0.75 | 0.05 |
| **AKI stage is 1,2** |  |  |  |  |  |
| HR (95%CI) | 1.53(1.20,1.96) | 1.31(1.09,1.57) | Reference | 1.07(0.84,1.38) | 1.22(0.71,2.09) |
| P value | <0.01 | <0.01 |  | 0.55 | 0.48 |
| **AKI stage is 3** |  |  |  |  |  |
| HR (95%CI) | 1.23(1.04,1.45) | 1.08(0.93,1.25) | Reference | 0.91(0.73,1.15) | 1.64(1.11,2.43) |
| P value | 0.01 | 0.31 |  | 0.43 | 0.01 |
| **SOFA ≥ 10** |  |  |  |  |  |
| HR (95%CI) | 1.34(0.96,1.35) | 1.11(0.95,1.30) | Reference | 0.66(0.48,0.91) | 1.18(0.72,1.94) |
| P value | 0.13 | 0.20 |  | 0.01 | 0.51 |
| **7≤SOFA<10** |  |  |  |  |  |
| HR (95%CI) | 1.81(1.35,2.45) | 1.13(0.90,1.42) | Reference | 1.25(0.93,1.67) | 2.25(1.30,3.92) |
| P value | <0.01 | 0.29 |  | 0.14 | <0.01 |
| **4≤SOFA<7** |  |  |  |  |  |
| HR (95%CI) | 2.49(1.61,3.86) | 1.45(1.12,1.88) | Reference | 1.11(0.81,1.53) | 1.09(0.46,2.54) |
| P value | <0.01 | <0.01 |  | 0.52 | 0.85 |
| **SOFA<4** |  |  |  |  |  |
| HR (95%CI) | 2.23(0.67,7.40) | 1.21(0.68,2.14) | Reference | 1.32(0.76,2.28) | 3.26(1.14,9.37) |
| P value | 0.19 | 0.52 |  | 0.32 | 0.03 |
| **SAPS II ≥ 40** |  |  |  |  |  |
| HR (95%CI) | 1.36(1.17,1.58) | 1.21(1.07,1.37) | Reference | 0.84(0.68,1.05) | 1.45(1.00,2.09) |
| P value | <0.01 | <0.01 |  | 0.12 | 0.05 |
| **SAPS II < 40** |  |  |  |  |  |
| HR (95%CI) | 1.61(1.11,2.33) | 1.12(0.88,1.42) | Reference | 1.35(1.03,1.77) | 1.57(0.83,2.97) |
| P value | 0.01 | 0.37 |  | 0.03 | 0.17 |

| **Table S3. Adjusted HR of hospital mortality according to BE among Subgroups** | | | | | |
| --- | --- | --- | --- | --- | --- |
| **Subgroups** | **BE groups(mEq/L)** | | | | |
|  | **BE≤-9 mEq/L** | **-9<BE≤-3 mEq/L** | **-3<BE≤3 mEq/L** | **3<BE≤9 mEq/L** | **BE>9 mEq/L** |
| **Male** |  |  |  |  |  |
| HR (95%CI) | 1.27(1.07,1.50) | 1.10(0.95,1.26) | Reference | 1.03(0.83,1.27) | 1.55(1.00,2.39) |
| P value | <0.01 | 0.20 |  | 0.80 | 0.05 |
| **Female** |  |  |  |  |  |
| HR (95%CI) | 1.28(1.06,1.54) | 1.10(0.95,1.28) | Reference | 1.00(0.81,1.23) | 1.63(1.12,2.36) |
| P value | 0.01 | 0.21 |  | 1.00 | 0.01 |
| **AKI stage is 1,2** |  |  |  |  |  |
| HR (95%CI) | 1.43(1.15,1.79) | 1.20(1.03,1.40) | Reference | 1.09(0.88,1.35) | 1.59(1.04,2.43) |
| P value | <0.01 | 0.02 |  | 0.42 | 0.03 |
| **AKI stage is 3** |  |  |  |  |  |
| HR (95%CI) | 1.19(1.02,1.39) | 1.04(0.91,1.19) | Reference | 0.95(0.77,1.17) | 1.64(1.12,2.39) |
| P value | 0.03 | 0.54 |  | 0.63 | 0.01 |
| **SOFA ≥ 10** |  |  |  |  |  |
| HR (95%CI) | 1.15(0.98,1.34) | 1.09(0.95,1.27) | Reference | 0.69(0.52,0.92) | 1.14(0.70,1.87) |
| P value | 0.09 | 0.23 |  | 0.01 | 0.60 |
| **7≤SOFA<10** |  |  |  |  |  |
| HR (95%CI) | 1.72(1.32,2.22) | 1.03(0.85,1.26) | Reference | 1.16(0.88,1.52) | 2.21(1.35,3.61) |
| P value | <0.01 | 0.75 |  | 0.29 | <0.01 |
| **4≤SOFA<7** |  |  |  |  |  |
| HR (95%CI) | 1.64(1.07,2.49) | 1.32(1.05,1.66) | Reference | 1.29(0.99,1.68) | 1.62(0.85,3.08) |
| P value | 0.02 | 0.02 |  | 0.06 | 0.14 |
| **SOFA<4** |  |  |  |  |  |
| HR (95%CI) | 1.35(0.42,4.35) | 1.14(0.70,1.86) | Reference | 1.11(0.68,1.81) | 2.82(1.23,6.47) |
| P value |  |  |  |  |  |
| **SAPS II ≥ 40** |  |  |  |  |  |
| HR (95%CI) | 1.29(1.13,1.48) | 1.15(1.03,1.29) | Reference | 0.89(0.73,1.07) | 1.53(1.10,2.14) |
| P value | <0.01 | 0.02 |  | 0.21 | 0.01 |
| **SAPS II < 40** |  |  |  |  |  |
| HR (95%CI) | 1.52(1.08,2.14) | 1.06(0.85,1.32) | reference | 1.30(1.02,1.66) | 1.98(1.18,3.35) |
| P value | 0.02 | 0.58 |  | 0.03 | 0.01 |

|  | **Table S4. Differences of LOS in ICU according to BE among Subgroups** | | | | |
| --- | --- | --- | --- | --- | --- |
| **Subgroups** | **BE≤-9 mEq/L** | **-9<BE≤-3 mEq/L** | **-3<BE≤3 mEq/L** | **3<BE≤9 mEq/L** | **BE>9 mEq/L** |
| **Male** |  |  |  |  |  |
| N | 747 | 1762 | 4834 | 812 | 106 |
| β | 0.21 | 0.40 | Reference | 0.43 | 0.51 |
| P value | 0.48 | 0.04 |  | 0.10 | 0.46 |
| **Female** |  |  |  |  |  |
| N | 622 | 1279 | 3203 | 731 | 142 |
| β | -1.70 | 0.07 | Reference | 0.53 | -0.25 |
| P value | 0.58 | 0.73 |  | 0.05 | 0.66 |
| **AKI stage is 1,2** |  |  |  |  |  |
| N | 597 | 1826 | 6039 | 1082 | 172 |
| β | 0.98 | 0.44 | Reference | 0.55 | 0.41 |
| P value | <0.01 | <0.01 |  | <0.01 | 0.32 |
| **AKI stage is 3** |  |  |  |  |  |
| N | 772 | 1215 | 1998 | 461 | 76 |
| β | -0.94 | -0.31 | Reference | 0.24 | -1.06 |
| P value | 0.03 | 0.36 |  | 0.61 | 0.33 |
| **SOFA ≥ 10** |  |  |  |  |  |
| N | 845 | 1111 | 1369 | 274 | 47 |
| β | 0.49 | 0.46 | Reference | -0.12 | 0.27 |
| P value | <0.01 | 0.23 |  | 0.21 | 0.75 |
| **7≤SOFA<10** |  |  |  |  |  |
| N | 208 | 839 | 2084 | 392 | 66 |
| β | -0.27 | -0.22 | Reference | 0.45 | 0.03 |
| P value | 0.51 | 0.41 |  | 0.21 | 0.97 |
| **4≤SOFA<7** |  |  |  |  |  |
| N | 191 | 788 | 2992 | 570 | 90 |
| β | -0.04 | 0.30 | Reference | 0.93 | -0.10 |
| P value | 0.94 | 0.22 | . | <0.01 | 0.88 |
| **SOFA<4** |  |  |  |  |  |
| N | 53 | 303 | 1592 | 307 | 45 |
| β | -0.77 | 0.06 | Reference | 0.35 | 0.19 |
| P value | 0.13 | 0.79 |  | 0.13 | 0.74 |
| **SAPS II ≥ 40** |  |  |  |  |  |
| N | 1066 | 1895 | 3584 | 719 | 115 |
| β | -0.15 | 0.04 | Reference | 0.33 | -0.14 |
| P value | 0.60 | 0.85 |  | 0.27 | 0.84 |
| **SAPS II < 40** |  |  |  |  |  |
| N | 303 | 1146 | 4453 | 824 | 133 |
| β | 0.18 | 0.29 | Reference | 0.67 | 0.35 |
| P value | 0.63 | 0.13 |  | <0.01 | 0.52 |

| **Table S5. Differences of LOS in hospital according to BE among Subgroups** | | | | | |
| --- | --- | --- | --- | --- | --- |
| **Subgroups** | **Median (IQR)** | | | | |
|  | **BE≤-9 mEq/L** | **-9<BE≤-3 mEq/L** | **-3<BE≤3 mEq/L** | **3<BE≤9 mEq/L** | **BE>9 mEq/L** |
| **Male** |  |  |  |  |  |
| N | 747 | 1762 | 4834 | 812 | 106 |
| β | -0.01 | 1.67 | Reference | 0.04 | 0.57 |
| P value | 0.99 | 0.01 |  | 0.94 | 0.68 |
| **Female** |  |  |  |  |  |
| N | 622 | 1279 | 3203 | 731 | 142 |
| β | 0.23 | -0.07 | Reference | 0.23 | -0.10 |
| P value | <0.01 | 0.67 |  | 0.85 | 0.61 |
| **AKI stage is 1,2** |  |  |  |  |  |
| N | 597 | 1826 | 6039 | 1082 | 172 |
| β | 1.54 | 1.17 | Reference | 5.67 | 1.42 |
| P value | <0.01 | <0.01 |  | 0.12 | 0.11 |
| **AKI stage is 3** |  |  |  |  |  |
| N | 772 | 1215 | 1998 | 461 | 76 |
| β | -1.25 | 0.43 | Reference | -1.04 | -3.00 |
| P value | 0.09 | 0.47 |  | 0.20 | 0.11 |
| **SOFA ≥ 10** |  |  |  |  |  |
| N | 845 | 1111 | 1369 | 274 | 47 |
| β | -0.57 | 0.62 | Reference | -0.44 | -1.93 |
| P value | 0.48 | 0.38 |  | 0.70 | 0.46 |
| **7≤SOFA<10** |  |  |  |  |  |
| N | 208 | 839 | 2084 | 392 | 66 |
| β | 0.40 | 0.72 | Reference | -0.63 | 0.70 |
| P value | 0.59 | 0.13 |  | 0.32 | 0.63 |
| **4≤SOFA<7** |  |  |  |  |  |
| N | 191 | 788 | 2992 | 570 | 90 |
| β | 1.13 | 1.08 | Reference | 0.60 | 0.17 |
| P value | 0.15 | <0.01 |  | 0.21 | 0.88 |
| **SOFA < 4** |  |  |  |  |  |
| N | 191 | 788 | 2992 | 570 | 90 |
| β | -0.03 | 0.73 | Reference | 0.91 | 1.84 |
| P value | 0.98 | 0.17 |  | 0.09 | 0.17 |
| **SAPS II ≥ 40** |  |  |  |  |  |
| N | 1066 | 1895 | 3584 | 719 | 115 |
| β | -0.26 | 0.44 | Reference | -0.60 | -0.20 |
| P value | 0.61 | 0.27 |  | 0.30 | 0.88 |
| **SAPS II < 40** |  |  |  |  |  |
| N | 303 | 1146 | 4453 | 824 | 133 |
| β | 0.80 | 1.55 | Reference | 0.86 | 0.70 |
| P value | 0.25 | <0.01 |  | 0.05 | 0.50 |

| **Table S6** **use of** **bicarbonate and lactate before ICU intime among BE groups** | | | | | | |
| --- | --- | --- | --- | --- | --- | --- |
|  | **BE≤-9** | **-9<BE≤-3** | **-3<BE≤3** | **3<BE≤9** | **BE>9** | **P value** |
|  | **N=1369** | **N=3041** | **N=8037** | **N=1543** | **N=248** |  |
| bicarbonate | 49 (3.58%) | 76 (2.50%) | 134 (1.67%) | 43 (2.79%) | 6 (2.42%) | <0.001 |
| lactate | 68 (4.97%) | 188 (6.18%) | 489 (6.08%) | 79 (5.12%) | 9 (3.63%) | 0.133 |

| **Table S7 HR of 30-day hospital mortality according to BE in patients with AKI** | | | | |
| --- | --- | --- | --- | --- |
| **BE value** | **N** | **events** | **HR(95%CI)** | **P** |
| BE≤-9 | 1369 | 389 | 1.29(1.13,1.47) | <0.01 |
| -9<BE≤-3 | 3041 | 584 | 1.12(1.01,1.24) | 0.04 |
| -3<BE≤3 | 8037 | 965 | Reference | |
| 3<BE≤9 | 1543 | 197 | 1.00(0.86,1.16) | 0.99 |
| BE>9 | 248 | 54 | 1.54(1.16,2.04) | <0.01 |
| Adjusted by gender, age, ethnicity, AKI stage, PCO2, Co-morbidities, urine output, SOFA, SAPSII, RRT, vasopressor, ventilation, Creatinine, bicarbonate, and lactate | | | | |


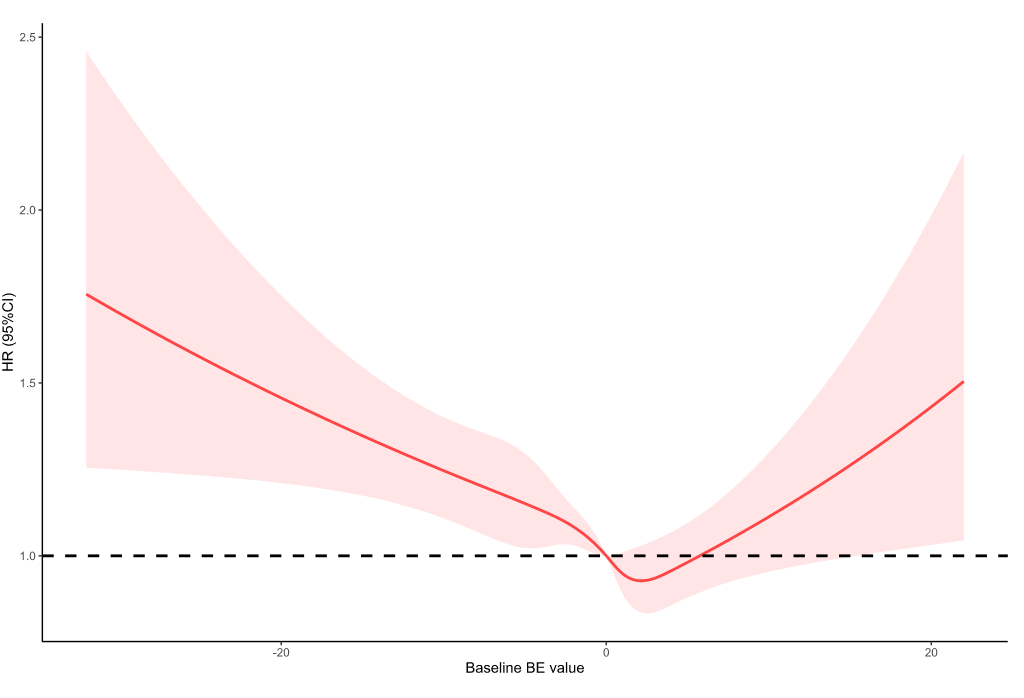


Figure S1. Baseline BE was modelled as a continuous variable and adjusted by gender, age, ethnicity, AKI stage, PCO2, Co-morbidities, urine output, SOFA, SAPSII, RRT, vasopressor, ventilation, Creatinine bicarbonate, and lactate.
